# Argonaute and Argonaute-Bound Small RNAs in Stem Cells

**DOI:** 10.3390/ijms17020208

**Published:** 2016-02-04

**Authors:** Lihong Zhai, Lin Wang, Feng Teng, Lanting Zhou, Wenjing Zhang, Juan Xiao, Ying Liu, Wenbin Deng

**Affiliations:** 1 Medical College, Hubei University of Arts and Science, Xiangyang 441053, Hubei, China; zhailihong@webmail.hzau.edu.cn (L.Z.); tengfeng1217@webmail.hzau.cn (F.T.); lanting_zhou@yeah.net (L.Z.); starking521@webmail.hzau.edu.cn (W.Z.); xiaojuan@bjmu.edu.cn (J.X.); 2 Xiangyang Oral Hospital, Xiangyang 441003, Hubei, China; tobenumberone@yeah.net; 3 Department of Neurosurgery, Medical School, the Brown Foundation Institute of Molecular Medicine for the Prevention of Human Diseases, University of Texas Health Science Center at Houston, Houston, TX 77030, USA; 4 Center for Stem Cell and Regenerative Medicine, the Brown Foundation Institute of Molecular Medicine for the Prevention of Human Diseases, University of Texas Health Science Center at Houston, Houston, TX 77030, USA; 5 Department of Biochemistry and Molecular Medicine, School of Medicine, University of California, Davis, CA 95817, USA; 6 Institute for Pediatric Regenerative Medicine, Shriners Hospitals for Children, Sacramento, CA 95817, USA

**Keywords:** Argonaute, Piwi, small RNAs, stem cells

## Abstract

Small RNAs are essential for a variety of cellular functions. Argonaute (AGO) proteins are associated with all of the different classes of small RNAs, and are indispensable in small RNA-mediated regulatory pathways. AGO proteins have been identified in various types of stem cells in diverse species from plants and animals. This review article highlights recent progress on how AGO proteins and AGO-bound small RNAs regulate the self-renewal and differentiation of distinct stem cell types, including pluripotent, germline, somatic, and cancer stem cells.

## 1. Introduction

Small RNAs, including microRNAs (miRNAs), short interfering RNAs (siRNAs) and Piwi-interacting RNAs (piRNAs), achieve their gene silencing functions at diverse levels of cellular progresses, including at the transcription, RNA processing, RNA stability, translation and post-translational levels [1,2]. These small RNAs execute their effects by associating with Argonaute (AGO) proteins, which are further divided into three subfamilies, AGO, Piwi, and Group 3 (known as WAGO) subfamilies. The miRNAs and siRNAs interact with AGOs, while piRNAs mainly interact with Piwi. Since Group 3 (WAGO) subfamily is only found in worms, we focus on AGO and Piwi in this review. Both protein subfamilies are found in a range of stem cell types and have important roles in a variety of cellular functions, including stem cell maturation and differentiation, embryonic development, and transposon functions [3], which critically contribute to the regulation of self-renewal, proliferation and differentiation of stem cells via specific mechanisms [4,5,6,7]. This concise review provides an update on recent progress regarding AGO and AGO-bound small RNAs and their regulation of various types of stem cells, such as pluripotent, germline, somatic, and cancer stem cells.

## 2. The Argonaute (AGO) Family

The AGO protein family was first identified in plants. The term AGO was derived from *ago1* in *Arabidopsis* [8]. Shortly after the identification of *ago1*, several additional AGO proteins were discovered in numerous organisms for their function in small RNA-directed silencing [9]. According to their functional domains, AGO proteins are subdivided into AGO proteins (the AGO clade) and Piwi proteins (the Piwi clade) [10] (Table 1). A prototype member of the AGO clade is *Arabidopsis thaliana* AGO1, while all Piwi proteins, share significant homology to *Drosophila melanogaster* Piwi [11]. AGO proteins function by cooperating with miRNAs or siRNAs and regulate gene-silencing at thepost-transcriptional level. Piwi proteins act by binding to piRNAs and exert their function through silencing of transposable elements [11,12,13]. Group 3 subfamily is only found in worms (*Caenorhabditis elegans*, known as WAGO), while AGO and Piwi subfamilies are found in a variety of species, such as plants, yeasts, flies, and mammals. The number of AGO proteins varies among different species, the fission yeast (*Schizosaccharomyces pombe*) has only one AGO protein, while the nematode worm has 27 different AGO proteins. However, the structure of AGO proteins is highly conserved. In the mouse, eight AGO proteins are categorized into two subfamilies: five to the AGO subfamily (simplified as AGO-like) and the remaining three to the Piwi subfamily. Similarly, in human, based on NCBI’s sequence alignment, four AGO proteins (*hAGO1/EIF2C1*, *hAGO2*/*EIF2C2*, *hAGO3*/*EIF2C3*, and *hAGO4/EIF2C4*) and four Piwi proteins (*Hiwi/Piwil1*, *Hili/Piwil2*, *Piwil3*, and *Hiwi2/Piwil4*) [14] have been identified. Previous expression profiling in mammalian cells revealed that the expression of AGO subfamily members can be found in all tissues ubiquitously, whereas expression of the Piwi proteins seems to be restricted to germ cells [4]. Recent reports, however, have recognized somatic Piwi as well [15]. A complete tissue array of the various AGO proteins in different tissues will provide a comprehensive gene expression profile. Such work may offer clues to reveal potential novel functions of AGO proteins.

**Figure 1:**
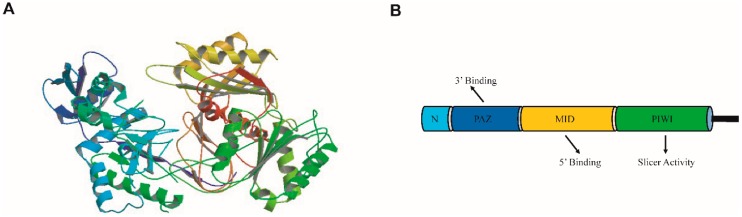
Domain schematics of a typical Argonaute protein. (**A**) Crystal structure of Aquifex aeolicus Argonaute (adapted from PDB database, PDB ID: 2NUB) [28], with stereo-view ribbon illustration of Argonaute showing the N-terminal domain (light blue), the PAZ domain (blue), the MID domain (yellow), the Piwi domain (green); (**B**) Schematic depiction of the Argonaute domain. PAZ domain anchors the characteristic 2-nt 3’ overhangs of small RNAs. The MID domain binds 5’ phosphates of small RNAs, and functions as an anchor of small RNAs binding to the Argonaute (AGO) protein. Piwi domain is an RNase H domain.

AGO proteins are large proteins and have a molecular weight of approximately 100 kDa. Regardless of the subfamilies, they usually contain one variable N-terminal domain, PAZ (Piwi-Argonaute-Zwille), MID (middle) and Piwi domains (Figure 1) [3]. The variable N-terminal region is reported to facilitate the separation of the small RNA: target duplex [9]. PAZ domain composes a particular binding module that anchors the 2-nt 3′ overhangs of small RNAs [3], which is critical for dsRNA processing in RNAi, as revealed by X-ray crystallography. The MID domain binds 5′ phosphates of small RNAs by a highly basic pocket, serving as an anchor of small RNAs onto AGO proteins [16,17]. Another function of the MID domain is to repress translation through a motif similar to eIF4E [18]. The Piwi domain contains a DDX (where X is D or H) motif, a catalytic triad DDX, which is homologous to RNase H enzymes and renders endonuclease activity for AGO proteins [19,20] (Figure 1). However, a DDX motif does not necessarily guarantee slicer activity [10] and only a subset of AGO proteins can cleave [11]. In the budding yeast *Kluyveromyces polysporus*, it seems that a constant glutamic acid (E) residue has to be tagged into the catalytic pocket to create a DEDD or DEDH tetrad for slicing activity [21]. In human, endonuclease activity of AGO2 might come from several aspects: the Piwi domain, a loop in the N terminal domain, and possibly unidentified AGO2 binding partners [22]. The 2.3 angstrom resolution crystal structure of human AGO2 has revealed that all the characteristic AGO domains are conserved in human AGO2, and that the interaction sites (tryptophan-binding pockets) in the Piwi domain for binding GW motif also exist [23]. QF-V motif of Piwi domain recognizes base-paring at the 15th base of a miRNA duplex, whereas recognition of the central nucleotides is critically dependent upon the DDDE catalytic core of AtAGO2 [24]. In addition to domains related to endonuclease activities, AGO protein motifs for small RNA sorting and protein-protein interactions have also been characterized in more depth. For instance, MID domain of human AGO2 has been reported to interact with fragile X mental retardation protein (FMRP) via a specific binding pocket composed of one tyrosine and two lysine residues, FMRP subsequently associates with miR-196a to silence HOXB8 mRNA [25]. Therefore, MID domain in AGO2 is critical for miR-196’s silencing effect toward HOXB8. Human AGO2 might also function as a framework by binding to guide RNA and target RNA duplex formed by pairing of miRNAs and the complementary target RNAs [26]. Another function of human AGO2 is to interact with A nucleotides through the water-mediated interaction of t1-nucleotide binding pocket and adenosine N6 amine, ensuring fixation of target RNAs on AGO2 [24,27].

**Table 1:** Characterized Argonaute (AGO) proteins in different species.

Gene Name(Subfamily)	Molecular Function	sRNA Bound	Reference
*Homo sapiens*			
*EIF2C1*/*hAGO1* (AGO)	miRNA-directed target gene regulation, constitutive and alternative splicing, heterochromatin silencing	miRNAs	[29,30,31]
*EIF2C2*/*hAGO2* (AGO)	miRNA-directed target gene regulation, heterochromatin silencing, RNAi	miRNAs, siRNA	[30,31,32]
*EIF2C3*/*hAGO3* (AGO)	miRNA-directed target gene regulation	miRNAs	[30]
*EIF2C4*/*hAGO4* (AGO)	miRNA-directed target gene regulation	miRNAs	[22]
*Piwil1*/*Hiwi* (Piwi)	stem cell self-renewal, division, gametogenesis, germ cell proliferation, and RNAi	piRNAs	[3,12]
*Piwil2*/*Hili* (Piwi)	signaling regulation	piRNAs	[33]
*Piwil3* (Piwi)	n/d	piRNAs	[3,12]
*Piwil4*/*Hiwi2* (Piwi)	transposon silencing	piRNAs	[34]
*Drosophila melanogaster*			
dAGO1 (AGO)	miRNA-mediated gene silencing	22–23-nt miRNAs	[35,36]
dAGO2 (AGO)	RNAi in embryos	miRNAs and 21-nt siRNAs	[35,36,37,38]
dAGO3 (Piwi)	transposon silencing	piRNAs	[39,40,41]
Aubergine/AUB (Piwi)	transposon silencing, stellate silencing, RNAi	piRNAs	[41,42]
Piwi (Piwi)	transposon silencing, germline stem-cell maintenance, RNAi	piRNAs, rasiRNAs	[41,42,43]
*Arabidopsis thaliana*			
AtAGO1	plant development regulation and stress responses	21-nt miRNAs	[8,44,45]
AtAGO2	antibacterial immunity, viral defense, DSB-induced sRNAs activity, and DNA repair	21-nt miRNAs, vsiRNAs, diRNAs	[46,47,48]
AtAGO3	n/d	n/d	
AtAGO4	RdDM pathway	24-nt siRNAs	[49,50]
AtAGO5	initiation of megagametogenesis, antiviral RNA silencing	siRNAs	[46,51,52,53]
AtAGO6	methylation of tasiRNA-generating loci and transcriptionally active TEs, shoot and root meristems	24-nt siRNAs, 21–22-nt endo-siRNAs	[49,54,55,56]
AtAGO7/ZIPPY	TAS3-derived tasiRNA biogenesis, leaf development	miR390	[57]
AtAGO8	Proposed to be a pseudogene	n/d	[10]
AtAGO9	germ cell fate repression in the somatic companion cells surrounding MMC, and DNA repair	24-siRNAs	[46,58]
AtAGO10/PINHEAD/ZWILLE	regulation of shoot apical meristems	miR165/166, miR172	[59,60,61,62,63]
*Oryza sativa*			
OsAGO1a/b/c	miRNA-directed target gene regulation	21-nt miRNAs	[64]
OsAGO5c/MEL1	regulation of cell division of premeiotic germ cells	21-nt siRNAs	[65,66]
OsAGO7	TAS3-derived tasiRNA biogenesis	miR390	[67]
OsAGO10/OsPNH1	regulates leaf development and maintenance of the shoot apical meristem	n/d	[68]
OsAGO18	broad-spectrum virus resistance	n/d	[69]
*Zea mays*			
ZmAGO7/RGD2	TAS3-derived tasiRNA biogenesis	miR390	[70]
ZmAGO9/AGO104	somatic cell fate repression in the germ cells	n/d	[71]
ZmAGO18b	tapetum and germ cell development	n/d	[72]

n/d, not determined.

## 3. The Association of AGO Proteins and Small RNAs

**Figure 2:**
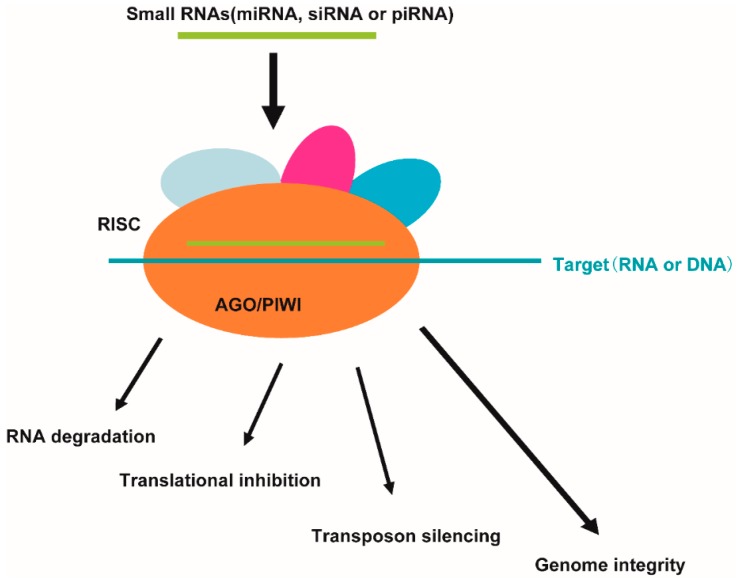
Guiding small RNAs’ gene silencing functions by AGO proteins.

RISC (RNA-induced silencing complexes). AGO proteins recruit specific small RNAs to form RISCs, which target specific sequences in RNA or DNA in the genome (Figure 2). All types of described small RNAs (namely miRNA, siRNA, and piRNA) are known to associate with AGO and/or Piwi proteins to form RISCs [73]. Small RNAs in RISCs serve as guides for AGOs, which execute enzyme functions at specific locations. Interestingly, to perform their effector functions, small RNAs must be incorporated into AGO-protein containing complexes [74,75]. Otherwise, they would be degraded rapidly in cells. Depending on the subcellular locations and AGO proteins associated within the complex, different RISCs will cause cleavage of target mRNAs, inhibit translations, or perform chromatin modification of target genes [76] (Figure 2). In miRNA pathway, only the guide strand of small RNAs will base-pair with the target mRNA and is retained, while the passenger strand is disposed and degraded [1]. Similarly, during the canonical siRNA pathway, RISC is brought to RNA targets by the siRNA guide strand, and the Piwi domain of the AGO proteins is responsible for precisely slicing target RNAs. Piwi domain cleaves at the phosphodiester bond at No. 10 and 11 nucleotides. The cleaved products will have 5′-monophosphate and 3′-hydroxyl termini [77]. In the case of an imperfect matching of siRNA and its target, two additional mechanisms are at work to silence the gene. One is through the posttranscriptional level, such as translational repression. The other is transcription gene silencing (TGS) by siRISCs via histone H3 or DNA methylation [1]. In addition to functioning in gene silencing, it is reported that *Arabidopsis* AGOs AtAGO2 and AtAGO9 might participate in DNA repair, although detailed mechanism of action remains to be unraveled [46].

**Figure 3:**
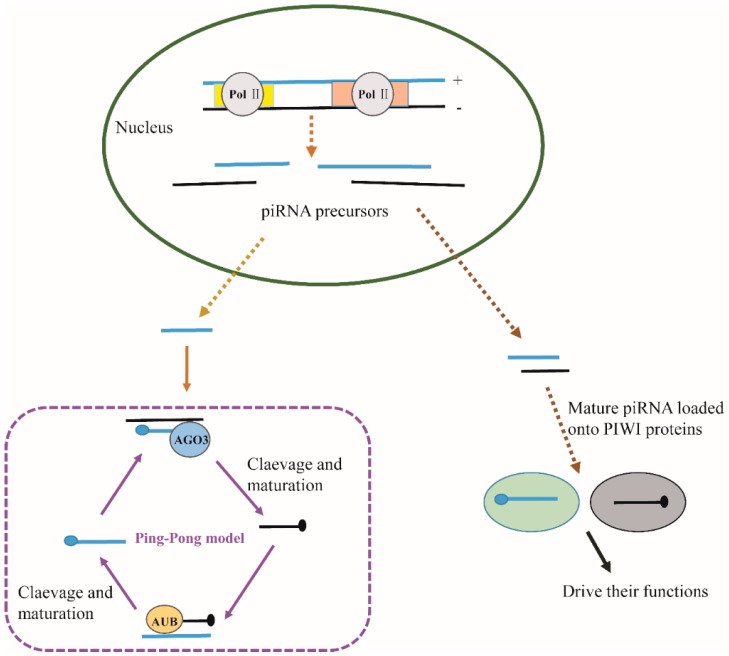
Biogenesis of piRNAs and the “Ping-Pong” model in *D. melenogaster*. piRNA clusters in the genome can be transcribed in either direction, sense or antisense. The long single-stranded RNA is the basis for piRNA generation. In *D. melanogaster*, to create sense and antisense piRNAs, Aubergine (AUB) and Ago3 two Piwi proteins, partner in secondary piRNA production. AGO3 only associates to piRNAs that are generated from retrotransposons’ sense strand. On the contrary, AUB mainly binds piRNAs made from the antisense strand. This phenomenon leads to the “Ping-Pong” model. Based on “Ping-Pong” model, AGO3-piRNA complex induced cleavage specifies the 5’-end of AUB-associated piRNAs, the AUB-piRNA complex then generates the 5’-end of AGO3-bound piRNAs. Mature piRNAs are loaded onto Piwi proteins and guide them to transposable elements derived complementary RNAs. Piwi proteins slice the transposon RNA to induce silencing in a similar fashion to RNAi.

Piwi and piRNAs. piRNAs are noncoding RNAs that bind Piwi proteins. Distinct from miRNAs and siRNAs, piRNAs have a length of 26–31 nt. piRNAs precursors are single stranded and are generated from repetitive sequences in the genome. Biogenesis and stability of piRNA might rely on Piwi proteins [18], as illustrated in the so-called “Ping-Pong” model (Figure 3), in which Piwi proteins cooperate in secondary piRNA production. piRNA-Piwi complexes also play important roles in maintaining genome integrity by preventing excessive transposon mobilization [78], which contributes to genome instability by replicating and/or inserting into new genomic locations. piRNA-Piwi complexes are proposed to act at various levels to control transposon activity: (1) At the post-transcription level, by destructing mRNA transcripts in the cytoplasm, one example is MIWI-piRNA, which functions in the mouse germline cells [43,79]. Another example is the regulation of maternal mRNA by piRNA and a newly identified Piwi protein, Aubergine (AUB) [80]; (2) at the transcription level, piRNA-Piwi interferes with heterochromatin assembly, which results in transcriptional silencing [81,82,83]; (3) at the translation level, piRNA-Piwi interacts with translation initiation factors, a classical example of which is the interaction of MILI, a mouse Piwi protein, with eIF3a and eIF4G [5]. In addition, piRNAs might regulate gene expression independent of Piwi proteins. For example, piR-55490 is reported to be able to bind mTOR mRNA directly and to induce its degradation [84].

Construction of AGO-small RNA target network. Although sharing similar functional domains, apparently AGO proteins have their own preference in choosing small RNA binding partners. Traditionally, quantitative real-time PCR, degradome sequencing and 5′ RLM-RACE (RNA Ligase Mediated Rapid Amplification of cDNA Ends), have been used in predicting AGO and small RNA target pairs [85,86,87]. The development of high-throughput sequencing allows for global profiling of RNAs isolated from immunopurification (RIP-seq) and by crosslinking and immunoprecipitation (CLIP-seq or HITS-CLIP), paving the way to identification of AGO protein–small RNA-mRNA interaction sites in both plants and animals [20,52,88,89,90,91]. Computational prediction of small RNA targets relies on a combinatorial approach. Rules frequently are derived from previously validated binding partners, and *in silico* prediction is usually the first step of providing clues of candidates for AGO and small RNA binding partners. Indeed, identification of the small RNAs sorting into the appropriate AGO proteins is a prerequisite for elucidating a small RNA’s function. Several observations have been made: (1) the size of small RNAs. Different *Arabidopsis* AGO (AtAGO) proteins bind small RNAs with distinct sizes. AtAGO1 or AtAGO2 is found to form complexes with small RNAs of 21 nt long, AtAGO4 and AtAGO9 with small RNAs of 24 nt in size. AtAGO5 is less selective as it binds small RNAs in the length of 21, 22 or 24 nt; (2) chemical composition/modification of termini. AtAGO1 binds small RNAs with a uridine at 5’, AtAGO2, AtAGO4 prefers small RNAs with an adenosine at 5’, and AtAGO5 binds small RNAs with a cytosine at 5’ [52]. AtAGO7 is more biased in choosing small RNAs and only forms interactions with miR390 [92]; (3) origin (precursors) of small RNAs. A good example is that AtAGO9 preferentially forms complexes with small RNAs derived from transposable elements (TEs) [93]. These observations have facilitated the development of protocols and computational algorithms for identification of AGO-small RNA network in higher organisms including mammals and humans. Additional more advanced approaches have also been developed. For instance, high-throughput sequencing of RNA extracted from crosslinking immunoprecipitation (HITS-CLIP) using highly selective monoclonal antibodies have identified additional AGO binding partners. In mouse testes, MIWI- and MILI-associated piRNAs are determined to be 29–31 or 26–28 nt in length, respectively [94,95], confirming the predicted sites from different computer algorithms [96], offering increased confidence in conducting AGO HITS-CLIP in other organisms [88,91,97,98]. CLIP-seq of AGO2 in mouse ESCs revealed that GCACUU motif in 3′UTRs and CDS correspond to miRNA seed matches [99]. Recently, a study of analyzing 34 AGO HITS-CLIP datasets observed that in different cell types, miRNA targets show distinct distribution patterns in the genomes, while in same cell types, the distributions are also identical [100]. Elucidation of AGO-small RNAs-targets network with HITS-CLIP in specific tissues or cells is revealing the regulatory mechanism of small RNAs.

## 4. Argonaute-Interacting Partners and Regulation of Argonaute Function

AGO-interacting proteins. Argonautes participate in many maturation processes of small RNAs as well as gene repression pathways that are mediated by small-RNAs, which involve interactions with diverse protein complexes. During miRNA-directed mRNA inhibition, the N-terminal domain of GW182 proteins, which contains multiple bi amino acid repeats of glycine-tryptophan (GW), that binds to AGO proteins. Such binding is necessary to facilitate all downstream gene silencing effects [101]. The C-terminal part of GW182, on the other hand, interacts with the poly (A)-binding protein on the poly (A) tail of the mRNA. In addition, direct interactions of the C-terminal part with PAN2/3 and the CCR4/NOT complex, which are cellular deadenylases, deadenylate the mRNA. Subsequently, the mRNA is decapped by DCAP1/2 and then degraded by the exonuclease XRN1 [102,103,104,105]. To make the process even more complex, other proteins also participate and regulate at additional levels. For instance, Pumilio (Pum) protein regulates the efficiency of miRNA-directed gene silencing [106], impotin8 (IPO8) or UPF1 maintains target association of AGO complexes [107,108], and UBR5 facilitates in recruiting downstream factors by associating with GW proteins [109]. There also have been reported some proteins interacting with Piwi-piRNA complexes. For example, protein arginine *N*-methyltransferase 5 (PRMT5), Tudor-domain-containing proteins (TDRDs) Tudor domain proteins Tud, spindle E (SpnE), krimper (Krimp), female sterile (1) Yb (Fs(1)Yb), tejas (Tej), vreteno (Vret), *etc*. However, the molecular mechanisms remain to be revealed [11]. Recently, CG9754/Silencio has been found to be an essential factor for Piwi-mediated transcriptional silencing in *Dropsophila* during heterochromatin formation [110].

Regulation of AGO function. Similar to majority genes, gene expression of AGO is also regulated at several levels. For example, AtAGO1 mRNA is found to be the target of miRNA168 and this process is needed for proper plant development, exemplifying the importance of feedback control by miRNA [44]. Similarly, the expression of human AGO2 was inhibited by miR-132 during primary cell activation [111], on the contrary, miR346 enhanced the expression of AGO2, leading to the increased activity of other miRNAs and contributing to the malignancy of Hela cells [112]. In addition to small-RNA directed gene silencing, ubiquitylation and phosphorylation were also identified to control the expression of AGO proteins [11].

## 5. AGO Proteins and AGO-Bound Small RNAs in Stem Cells

**Figure 4:**
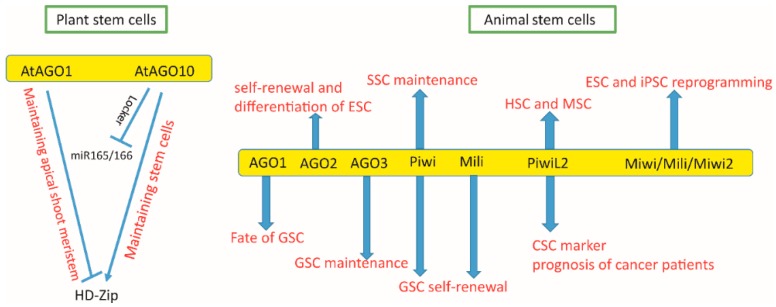
Summary of Argonaute proteins function in various stem cells.

AGO proteins and AGO-bound small RNAs are identified in various types of stem cells [113] in diverse species ranging from plants and animals (Figure 4). The AGO family was first identified to be essential for stem cell maintenance in lower organisms, such as *Drosophila*, *C. elegans*, and *Arabidopsis* [114], as knocking out AGO proteins decreases the number of stem cells or abolishes normal development [4]. To date, all three AGO subfamilies have been reported to function in various stem cell types, including germline and non-germline stem cells, normal and malignant stem cells, and pluripotent and somatic stem cells. Here, we emphasize the expression and function of the AGO/Piwi family in plant stem cells, germline stem cells, pluripotent stem cells (including embryonic stem cells andinduced pluripotent stem cells), somatic stem cells (including hematopoietic stem cells and mesenchymal stem cells), as well as cancer stem cells.

Plant stem cells. Plant stem cells are found at the tips of roots, shoots, and developing flowers. Stem cells located at different organs of a plant have distinct functions. The above-ground portion of the plant is derived from the stem cells in the shoot apical meristem (SAM) [63]. The stem cells from the meristem are committed to becoming a single central organ of the plant [4]. Loss of function mutation of AtAGO1 (named for *Arabidopsis* AGO, or PINHEAD/ZWILLE in mammals), exhibits phenotypes of both abnormal axillary meristems and leaf formation, indicating that *AtAGO1* is essential in SAM function [8]. On the other hand, AtAGO10 (PINHEAD/ZWILLE) is required for shoot meristem formation via self-perpetuating shoot meristem divisions [59]. Apparently AtAGO1 and AtAGO10 have functional redundancy because only double mutants demonstrate impaired SAM initiation and maintenance, which act through a pathway that is regulated by SHOOT MERISTEMLESS, a homeobox transcription factor [60]. However, AtAGO1 and AtAGO10 act via distinct mechanisms. AtAGO1 mainly functions through associating with miRNAs and siRNAs, and its effector complexes cleave mRNAs via slicer activity [115]. However, AtAGO10 exerts regulatory functions through miRNA-mediated translational repression [116]. It appears that AtAGO1 and AtAGO10 are both important in stem cells’ temporal control. Both AGO proteins repress AP2 and HD-Zip through miRNA miR172 and miR165/166, It is interesting to note that AtAGO1 and AtAGO10 function in opposite ways both in accumulation of miR165/166 and in controlling the gene expression of HD-Zip genes. In *ago10* mutants, the expression of miR165/166 is increased and the expression of HD-Zip genes is decreased. This suggests that AGO10 upregulates HD-Zip expression via decreasing miR165/166 levels by acting as a locker for miR165/166. Interestingly, AGO1 and AGO10 do not always function in different directions as in HD-Zip gene expression regulation, and they actually both boost floral determinacy. One possible explanation is that up- and down-regulation of different HD-Zip genes are involved in floral determinacy [62,63].

**Figure 5:**
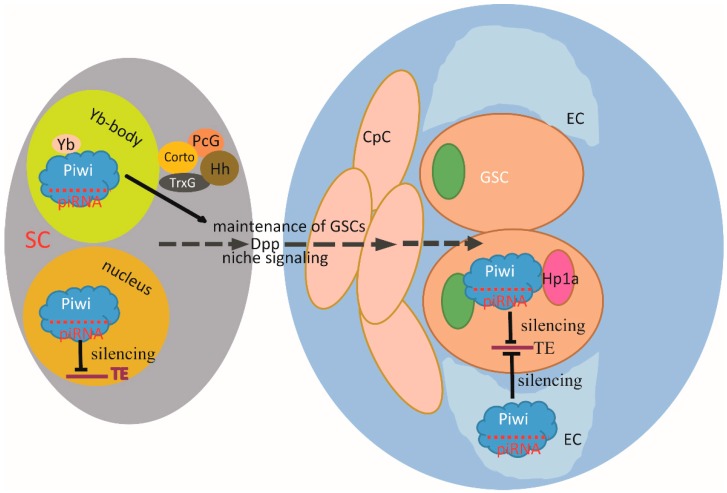
Piwi function in Drosophila GSCs self-renewal. Stem cell self-renewal requires both cell-autonomous and extrinsic signaling mechanisms. Niche signaling pathways involving Yb, Piwi, Dpp and Hh are expressed in SCs for GSC maintenance. The chromatin factor Corto, which is expressed in the niche cells, interacts with Piwi, PcG and TrxG in epigenetic regulation. Piwi also interacts with HP1a for TEs silencing for GSCs self-renewal. Piwi in ECs maintains PGC and GSC germlines and controls GSC lineage differentiation. Dpp: Decapentaplegic; Hh: Hedgehog; SCs: Somatic Cells; GSC: Germ Stem Cell; PcG: Polycomb group; TrxG: Trithorax group; HP1a: Heterochromatin protein 1a. TEs: Transposable elements; ECs: Escort cells; PGC: Primordial germ cell.

Germline stem cells. *Drosophila* Piwi family member, *Piwi*, is expressed in both the germline and soma. Piwi is essential for self-renewal and asymmetric division GSCs [114]. Both male and female *Piwi* mutant flies are infertile, are depleted of GSCs, and their gonads contain only significantly lower number of egg chambers or sperm bundles. Piwi maintains GSCs and controls their differentiation in both cell-autonomous and non-cell-autonomous manners [114]. Somatic Piwi provides a niche to GSCs by upregulating decapentaplegic (Dpp) pathway [117,118]. *Piwi* also interacts with *Corto*, a gene encodes a chromatin protein, which controls *Piwi* expression in niche cells to maintain GSCs through an epigenetic mechanism. On the other hand, loss of *Piwi* also causes GSC-like tumors that persist throughout adult life and defects in intermingled cells (ICs), the progenitor cells of escort cells and escort cells, causing ectopic Dpp signaling and subsequent prevention of GSC differentiation [118]. Previous research has shown that *Drosophila* *Piwi* functions on genome stability maintenance in the germline. Piwi silences transposable elements (TEs) by recruiting ~26–32 nt piRNAs [119]. However, more recent data from a *Piwi^Nt^* mutation, which is devoid of the nuclear localization signal (NLS), show that female flies could maintain self-renewal of the GSCs. The number of egg chambers is not affected either. The only phenotype is the derepression of transposable elements (TE) and nuclear accumulation of their mRNAs in the germ line. These results indicate that *Piwi* controls self-renewal of GSCs via GSC niche cells but not through manipulating TE function [120] (Figure 5). In parallel, *Piwi* may contribute to the differentiation of GSCs via escort cells (ECs) [121]. In addition, *Piwi* also interacts with Fasciclin 3, a gene essential for somatic cyst cells in gonadal development, in a cell-autonomous fashion. Such interaction has added a novel layer of Piwi regulation in GSCs [15]. Not only *Piwi*, but also *Ago1* in *Drosophila* plays indispensable roles in regulating GSC fate. Overexpression of *Ago1* causes GSCs over-proliferation, whereas *Ago1* knockout results in GSC depletion. Unlike Piwi, AGO1, although also plays a role in GSC fate regulation, does not act upstream of “bag of marbles” (*bam*) and is dispensable for bam silencing. Current data indicates that AGO1 might function either downstream of bam or in parallel with *bam* [122]. In *ago3* mutant flies, GSCs are not properly maintained, thus causing reduced fertility [123].

Mice have three Piwi proteins, Mili (PiwiL2), Miwi, and Miwi2, all of which are essential for spermatogenesis. Mili is detected in the cytoplasm of spermatogonia, testicular GSCs, and chromatoid bodies of early spermatocytes it is. Mili loss-of-function mutants show failure of self-renewal and differentiation of GSCs. However, the primary germ line stem cell population is not affected. Interestingly, *Mili* mutation decreases the turnover of protein synthesis of stem cell specific genes although does not function at the mRNA transcripts, suggesting a function of Mili in promoting GSC division and differentiation via translational regulation [5].

Cancer stem cells. Cancer stem cells (CSCs) are regarded as a sustainable source of malignant cells. These cells, though constitute an extremely low proportion of the total tumor cells, may serve as the culprit contributing to drug resistance, tumor recurrence, metastasis and progression [124]. Similar to other types of stem cells, CSCs self-renew and have the capacity to differentiate. Several Piwi family members have been found in different types of cancers. For example, PiwiL2 is expressed in precancerous stem cells and breast cancers. PiwiL2 expression levels correlate with breast stages and is a good candidate for diagnostic marker [125] and a therapeutic target [126]. Mili also is expressed in CSCs and has been reported to contribute to tumor initiation, progression and metastasis [126]. For example, piR-932 and Mili have been proposed to be positive regulators in the process of breast CSCs. Specifically, Mili and piR-932 promote the methylation of Latexin, which possesses tumor suppressor functions. Methylation of Latexin will silence the Latexin gene and therefore permitting cancer metastasis. Therefore, both piR932 and Mili may serve as potential therapeutic targets for preventing breast cancer metastasis of [127].

Several human Piwi proteins have been shown to be, responsible for CSC self-renewal and cancer metastasis in a wide range of cancer types. Increased expression of Hiwi has been correlated with a poor prognosis for patients with soft-tissue sarcomas (STS). Another human Piwi protein is Hili, and its expression has been correlated with pluripotent genes Oct4 and SOX2 in cancer tissues in colorectal cancer [128]. Hiwi also seems to play a key role in proliferation and metastasis of hepatocellular carcinoma (HCC) [129]. Hiwi also may facilitate chemoresistance in cervical cancer [130]. Further investigation on the function of AGO/Piwi proteins in human cancers might help identify additional tumor markers and drug targets [131].

Somatic stem cells. Somatic stem cells (SSCs) are found in unique stem cell niches located at different adult tissues. CSCs are lineage specific, have limited proliferation and differentiation capacity, and are only committed to certain specific cell fates. One example of SSCs is CD34^+^ hematopoietic stem cells (HSCs), which represent probably one of the best characterized SSCs. Hiwi has been identified to be expressed in human hematopoietic progenitor cells, but not in mature blood cells [132]. Miwi2 (Piwil4) is expressed in primitive hematopoietic cells. Interestingly, mice with a triple knockout of Miwi, Mili, and Miwi2, are able to maintain hematopoiesis in long-term [133], indicating their function is not essential for HSCs. Another example of Piwi’s function in SSCs is the requirement of Piwi in somatic cyst stem cell, which is essential for early germ cell maintenance. Because of the indispensable role of Piwi in GSCs, Piwi might serve as an important connection between the somatic and germline stem cell lineages [15]. Another example of SSCs is mesenchymal stem cells (MSCs). These SSCs potentially can robustly participate in tissue regeneration and repair [134]. Piwi protein PiwiL2 and associated piRNAs are expressed in bone marrow derived MSCs and may regulate the cell cycle of MSCs [135].

Pluripotent stem cells. Pluripotent stem cells (PSCs) include embryonic stem cells (ESCs) and induced pluripotent stem cells (iPSCs). Three Piwi genes, Miwi, Mili, and Miwi2, have been shown to be highly expressed in ESCs when compared to fibroblasts. However, mice knocking out all three Piwi genes are viable. Female knockout mice are able to breed although male mice are sterile. Miwi encodes a cytoplasmic protein specifically expresses in spermatocytes and spermatids [136]. Miwi2 is a nuclear protein expresses in the embryonic and neonatal stages. Miwi2 may function to control chromatin signatures of mESCs [137]. Furthermore, iPSCs are obtained at comparable efficiency from fibroblasts of triple knockout mice of Miwi/Mili/Miwi2 genes, indicating that at least these three Piwi proteins are dispensable iPSC reprogram process [138]. In human, AGO2 has been revealed to be indispensable for self-renewal and differentiation of ESCs by both siRNA and miRNA pathways through the research on Ago2-deficient ESCs [139]. Moreover, immunoprecipitation of human AGO2 has identified pluripotency-specific miRNAs expressed in both hESCs and iPSCs [32].

Four AGO proteins (AGO1-4) have been identified in mammals. AGO2 is the only one that has endonucleolytic activity. Oocytes from Ago2-deficient mice are able to mature, but have abnormal spindles and chromosomes, which prevent them from being able to cluster [140]. Female mice express Ago2^ADH^, a catalytically deficient knock-in allele of Ago2, which lack the function of endo-siRNAs, are infertile due to defects in meiosis I, indicating that catalytic activity of Ago2 is a prerequisite for meiosis in mouse oocytes to complete. Furthermore, some retrotransposon levels have been shown to be upregulated in Ago2^ADH^ oocytes [141]. Similar to mammal AGO2, Drosophila ago-2 embryos are defective in chromosome condensation, nuclear kinesis, and spindle apparatus assembly [142]. Therefore, AGO/Piwi protein bound small RNAs (miRNAs, siRNAs and piRNAs) are important players in ESCs through TE silencing for the proper formation of chromosomal structures, as well as gene expression regulation for normal metabolism.

## 6. Conclusions

The AGO protein family binds specific small RNAs in various tissues and influence stem cell properties including self-renewal, proliferation and differentiation, at several levels of gene expression regulation, including transcriptional and posttranscriptional repression, transposon and retro-transposon silencing, chromosomal modification. The relationship between AGO and its interacting proteins in gene regulation needed to be further defined. Construction of the regulation network of AGO-small RNAs-targets from various stem cells would be better revealed the AGO and small RNAs function. Systematic analysis on AGO modifications and regulations in stem cells are warranted, which will provide useful clues for stem cell biology and regenerative medicine, as well as for development of therapeutics for cancer patients.
